# Executive function is necessary for the regulation of the stepping activity when stepping in place in older adults

**DOI:** 10.1007/s40520-015-0499-9

**Published:** 2015-11-25

**Authors:** Christopher Dalton, Ria Sciadas, Julie Nantel

**Affiliations:** Faculty of Health Sciences, School of Human Kinetics, Ottawa University, 125 rue Université, Pavillon Montpetit, MNT 353, Ottawa, K1N 6N5 Canada

**Keywords:** Rhythmicity, Symmetry, Executive function, Older adults, Stepping, Gait

## Abstract

To determine the effect of age on stepping performance and to compare the cognitive demand required to regulate repetitive stepping between older and younger adults while performing a stepping in place task (SIP). Fourteen younger (25.4 ± 6.5) and 15 older adults (71.0 ± 9.0) participated in this study. They performed a seated category fluency task and Stroop test, followed by a 60 s SIP task. Following this, both the cognitive and motor tasks were performed simultaneously. We assessed cognitive performance, SIP cycle duration, asymmetry, and arrhythmicity. Compared to younger adults, older adults had larger SIP arrhythmicity both as a single task and when combined with the Category (*p* < 0.001) and Stroop (*p* < 0.01) tasks. Older adults also had larger arrhythmicity when dual tasking compared to SIP alone (*p* < 0.001). Older adults showed greater SIP asymmetry when combined with Category (*p* = 0.006) and Stroop (*p* = 0.06) tasks. Finally, they had lower cognitive performance than younger adults in both single and dual tasks (*p* < 0.01). Age and type of cognitive task performed with the motor task affected different components of stepping. While SIP arrhythmicity was larger for all conditions in older compared to younger adults, cycle duration was not different, and asymmetry tended to be larger during SIP when paired with a verbal fluency task. SIP does not require a high level of control for dynamic stability, therefore demonstrating that higher-level executive function is necessary for the regulation of stepping activity independently of the regulation of postural balance. Furthermore, older adults may lack the cognitive resources needed to adequately regulate stepping activity while performing a cognitive task relying on the executive function.

## Background

Gait is considered a highly complex motor task, as postural balance must be regulated to achieve safe forward progression while also coordinating symmetrical actions of both lower limbs. Normal gait pattern requires that movements of the legs be similar in amplitude and timing [1–3]. However, healthy individuals walk with some spatial and temporal variability [1], which may reflect the flexibility and adaptability of the motor system in the regulation of the gait pattern [4]. Others have suggested that some gait characteristics such as stride-to-stride variability should be kept low to maintain gait stability [5, 6]. Supporting this suggestion is the notion that gait variability and asymmetry tend to increase with advancing age and in the presence of neurological deficits (e.g. Parkinson’s disease) [7–9] and have been associated with a higher risk for falls in these populations [2, 10–14].

In younger adults, gait demands only minimal attention [15–17]. However, increasing evidence shows that gait regulation requires higher cognitive control, especially in older adults [18–20]. With advancing age, the ability to coordinate the action of each leg relative to the other becomes increasingly challenging, compromising gait symmetry, coordination, and ultimately reducing postural balance and increasing the risks for falls [17, 21]. Therefore, gait in older adults may not be as automatically regulated as it is in younger adults and may require more active control [17, 22, 23].

Dual-task paradigms have frequently been used to determine the cognitive processes involved in gait and often lead to a decrease in gait performance [15, 23–25] and an increased fall risk [17, 26]. Stride-to-stride variability has been found to be a reliable marker of gait regulation, where greater stride-to-stride variability represents poorer gait control as can be seen when two simultaneous tasks compete for one’s limited available attentional resources. Therefore, dual tasking involves the appropriate allocation and prioritization of attention between the two competing tasks [15, 23, 24, 27]. Typically, as the complexity of a cognitive task increases, the majority of attentional resources are directed towards the postural task to maintain motor performance and minimize postural instability [15, 28]. When the cognitive task is simple, the threat of postural instability is low, but as task difficulty increases, so does the postural threat until prioritization between the two tasks is required [15, 28]. As postural task complexity increases, older adults devote greater attention to the postural task compared to younger adults [18, 27–29].

As opposed to focusing on the entirety of a typical gait pattern, stepping-in-place (SIP) is a cyclical task, which reduces gait to its simplest form, as it does not include forward progression. Therefore, it reduces the need to regulate postural balance, and allows primarily assessing the regulation of the stepping action. SIP has been validated [30] and used as a surrogate to gait to assess stepping variability in individuals with Parkinson’s disease [10, 30] and subsequently in virtual reality protocols in PD [31, 32].

The purposes of the present study are to determine the effect of age on stepping variability and to compare the cognitive demand required to regulate repetitive stepping when performing a simple SIP task. More specifically, this study aims to determine the effects of two cognitive tasks relying on executive processes, the Category task and the Stroop Word and Color Test, to compare their impact on stepping variability between younger and older adults. Our hypothesis is that age will have an impact on stepping performance and that concurrently performing a cognitive task while SIP will have a greater effect in older adults compared to younger adults.

## Methods

Convenience sample of 29 subjects: 14 healthy younger adults (mean 25.4, SD 6.5, min 20, max 42 years, 11 women) and 15 healthy older adults (mean 71.0, SD 9.0, min 60, max 81 years, 10 women) participated in the study. Subjects were excluded if they reported previous surgeries (i.e. knee or hip replacement), impairment (i.e. knee or back pain) or conditions (i.e. Type 2 diabetes) that may interfere with gait and balance. None of the recruited participants were actually excluded as participants were screened during a telephonic interview performed prior to testing. Subjects were excluded if they reported previous surgeries and/or impairments (e.g. knee or back pain) that could result in proprioceptive alteration (e.g. knee or hip replacement), or any conditions such as Type 2 diabetes that could result in peripheral neuropathy and therefore interfere with gait and balance. Participants with uncorrected vision or vestibular problems were also excluded from the study. None of the recruited participants were actually excluded as they were screened during a telephonic interview performed prior to testing. Following this pre-screening, participants performed the Montreal Cognitive Assessment (MoCA) and were excluded if they presented with potential Mild Cognitive Impairment, i.e. if they had a score below 26. The study was approved by our Institutional Review Board. *Motor task* Participants were asked to stand upright with their arms along their sides and step in place at a comfortable pace for 60 s [30]. SIP trials, were performed on two force platforms recording at 200 Hz (Kistler, Winterthur, Switzerland). Different from Nantel et al. [30], SIP cycle duration, symmetry and rhythmicity were calculated for 55 s, to account for the non-steady state of the first few steps. SIP cycle asymmetry = 100 × |ln(SSWT/LSWT)|, where SSWT and LSWT correspond to the leg with the shortest and longest mean swing time over the trials, respectively. SIP cycle rhythmicity represents the mean stride time coefficient of variation (CV) of both legs. A large stride time CV indicates less rhythmic gait. SIP rhythmicity and symmetry were averaged over three trials. Swing and stride times were analyzed using the algorithm by Nantel et al. [30]. Force plate data were filtered with a zero-lag fourth-order Butterworth filter with a 12 Hz cut-off frequency.

*Cognitive tasks* The Category task and the Stroop Word and Color Test were performed while sitting (single task condition) and while SIP (dual task condition). The categories were presented on a monitor placed 1 m in front of the participant. The total length of the trial was 60 s, and each trial comprised of four categories (15 s each). The task consisted of naming as many items as possible that fit the given category in 15 s (e.g. naming as many vegetables as possible). Errors (e.g. naming a fruit in the vegetable category) were subtracted from the total number of items and categories were randomized between the sitting and SIP conditions. The Stroop Word and Color Test consisted of two parts: Stroop A (words) and Stroop B (colors). During the first part (Stroop A), participants were presented with an 8 × 10 matrix with color names written in different colored “inks”. Participants had 30 s to read the color names out loud regardless of the color of the “ink” (e.g. blue written in red = blue.). In the second part (Stroop B), a second matrix was presented again for 30 s, and participants were instructed to recite the color of the letters independently of the written word (e.g. blue written in red = red.). This task assesses the executive function by looking at the ability to appropriately allocate attentional resources and resolve conflict, unlike the category test, which involves mostly semantic memory and executive functions such as flexibility and inhibition [33]. *Variables* For the Category test, the total number of responses was the main variable while for the Stroop Word and Color Test, the main variables included the total number of words, total number of colors, as well as both the word–color absolute and relative differences.

### Statistics

Two-Way Mixed Design Analyses of Variance (ANOVAs) were used to account for the differences between age groups (younger and older adults) and attention (sitting and SIP) for cognitive performance (Category and Stroop tests). Two-Way Mixed Design ANOVAs were also used to compared SIP cycle duration, symmetry and rhythmicity between groups and between conditions (single or dual tasking). Repeated measures ANOVAs were also used to determine the difference between conditions within each group. Statistical level of significance was set at *p* < 0.05. All significant results were subjected to Bonferroni adjustment for multiple comparisons.

## Results

### Motor performance

For SIP arrhythmicity (Table 1), there was a significant main effect for tasks, *F*(2, 26) = 3.685, *p* < 0.001. Pairwise comparisons revealed a statistical difference between SIP as a single task *F*(1, 27) = 13.022, *p* = 0.001 and SIP combined with the Category task, *F*(1, 27) = 2.638, *p* = 0.001, and the Stroop Word and Color Test, *F*(1, 27) = 8.752, *p* = 0.006. There was also a main effect for groups, *F*(1, 27) = 17.814, *p* < 0.001. Older adults had significantly larger SIP arrhythmicity compared to younger adults when performing the SIP task as single task as well as when performed in combination with both the Category (*p* = 0.001) and Stroop Word and Color Test (*p* < 0.01), Table 1. Within the older adult group, SIP arrhythmicity during single tasking was smaller than during the Category and the Stroop Word and Color Test (*p* < 0.001). Also, when comparing dual task conditions, SIP arrhythmicity was smaller during the Stroop Word and Color Test compared to the Category test (*p* = 0.02). No differences were seen within the younger adult group.

**Table 1:** Asymmetry and arrythmicity (mean ± SD) as a single task and combined with the Category task and the Stroop Color–Word Test

	Young adults (*n* = 14)			Older adults (*n* = 15)		
	Single task	Category	Stroop	Single task	Category	Stroop
Arryhthmicity (CV)	2.63 ± 0.61	3.65 ± 0.87	3.53 ± 0.60	3.89 ± 1.16^†^	6.27 ± 2.62^†‡^	5.20 ± 2.04^†‡ω^
Asymmetry	4.13 ± 1.56	4.88 ± 2.47	4.73 ± 2.83	4.61 ± 1.79	6.93 ± 3.16^‡^	6.09 ± 2.52
Stepping in place cycle (s)	1.15 ± 0.14	1.18 ± 0.15	1.17 ± 0.14	1.13 ± 0.21	1.18 ± 0.23	1.14 ± 0.24

^†^Significantly different from young *p* < 0.01

^‡^Significantly different from single task *p* < 0.01

^ω^Significantly different from category task *p* < 0.05

In SIP asymmetry, there was a main effect for tasks, *F*(2, 26) = 3.869, *p* = 0.03. Pairwise comparisons revealed a difference between SIP as a single task and SIP combined with the Category task (*p* = 0.03). Within the older adult group, asymmetry of SIP alone was smaller compared to SIP with the Category task (*p* = 0.006) and a trend was seen with the Stroop Word and Color Test (*p* = 0.06). No differences were seen within the younger adult group.

No main effects were seen when comparing SIP asymmetry between groups, *F*(1, 27) = 3.503, *p* = 0.072. However, results showed a trend for statistical difference between groups in the Category task (*p* = 0.06). SIP stride duration in single or dual tasking was not significantly different between the groups or tasks.

### Cognitive performance

In the Category task, the total number of items named showed a main effect for tasks, *F*(1, 27) = 7.029, *p* = 0.01, as well as a main effect for groups *F*(1, 27) = 15.962, *p* < 0.001. Older adults named fewer items compared to the younger group for both single tasking, *F*(1, 27) = 8.169, *p* = 0.008, and dual tasking, *F*(1, 27) = 20.499, *p* < 0.001. Within the older adult group, the total number of responses during the Category test was lower when SIP (mean 27.5, SD 4.9) compared to when seated (mean 30.8, SD 7.3, *p* < 0.05). There were no differences between single (mean 37.3, SD 4.2) and dual (mean 35.8, SD 5.0) tasks in younger adults.

For the Stroop Word and Color Test, the number of words revealed a main effect for task, *F*(1, 27) = 17.708, *p* < 0.001, as well as for groups, *F*(1, 27) = 13.348, *p* = 0.001, Table 2. Pairwise comparisons revealed a difference between groups, with older adults identifying fewer items compared to younger adults when single tasking, *F*(1, 27) = 7.162, *p* = 0.01, and when dual tasking, *F*(1, 27) = 13.236, *p* = 0.001. Within the older adult group, fewer words were identified during dual tasking compared to single tasking (*p* < 0.01). No differences were seen between single and dual tasking conditions in younger adults.

**Table 2:** Stroop Color–Word Test (mean ± SD) while sitting (single task) and when combined with the SIP task

	Young adults (*n* = 14)		Older adults (*n* = 15)	
	Single task	Dual task	Single task	Dual task
Number of words	66.9 ± 9.6	60.4 ± 9.5	56.7 ± 10.8^†^	47.1 ± 10.1^†‡^
Number of colours	34.4 ± 4.2	36.4 ± 5.2	19.1 ± 7.2^†^	19.3 ± 7.7^†^
Difference (words–colours)	32.5 ± 9.2	24.1 ± 7.8^‡^	37.6 ± 12.8	27.8 ± 8.6^‡^
Colours/words (%)	52.1 ± 8.0	60.9 ± 9.0^‡^	34.7 ± 13.7^†^	40.9 ± 13.9^†‡^

^†^Significantly different from young *p* < 0.05

^‡^Significantly different from single task *p* < 0.05

The number of colors showed no main effect for task. However, there was a main effect for groups, *F*(1, 27) = 57.245, *p* < 0.001. Pairwise comparisons revealed fewer colors identified by older adults compared to younger adults both in single, *F*(1, 27) = 48.212, *p* < 0.001, and dual tasking conditions *F*(1, 27) = 48.079, *p* < 0.001.

The Stroop Word and Color Test difference showed a main effect for task, *F*(1, 27) = 16.390, *p* < 0.001. Both older (*p* < 0.001) and younger adults (*p* = 0.03) showed a larger word–colors difference in dual tasking compared to single tasking. The Stroop Word and Color Test difference ratio showed a main effect for task, *F*(1, 27) = 9.453, *p* = 0.005, and a main effect for groups, *F*(1, 27) = 27.956, *p* < 0.001. Pairwise comparisons showed smaller ratio in older adults compared to younger adults both in the single task, *F*(1, 27) = 17.127, *p* < 0.001, and dual task *F*(1, 27) = 20.656, *p* < 0.001. Within the older adults, the Stroop Word and Color Test difference ratio was larger in dual tasking (0.41 ± 0.03) compared to single tasking (0.35 ± 0.03), *p* < 0.001. Within the younger adults, the ratio was also larger in dual tasking (0.61 ± 0.03) compared to single tasking (0.52 ± 0.03), *p* < 0.001.

## Discussion

The main purposes of this study were to determine the effect of age on motor and cognitive performance and the impact of a two cognitive tasks relying on the executive function, the Category verbal fluency task and the Stroop Word and Color Test, on SIP regulation. Contrary to previous studies assessing the effect of age and dual tasking on gait variability, we chose the SIP task as it does not require control of the center of mass for forward gait progression and therefore allows for sole assessment of the stepping activity generation and regulation. As expected, our results showed that both motor and cognitive performances were affected by age. More importantly, we found that age and the type of cognitive task performed along with the motor task had an effect on different components of gait and cognitive performance. While arrhythmicity was larger in all conditions in older compared to younger adults, SIP cycle duration was not significantly different in any of the conditions. A trend toward larger asymmetry was found with older adults (*p* = 0.06), but only when SIP was combined with the Category task, not the Stroop.

The similar SIP cycle duration between groups and conditions is different from other studies which found slower gait speed in older adults especially when dual tasking. During normal walking, the progression of the center of mass within the margin of stability needs to be actively controlled by the central nervous system. Therefore, a reduction in speed when gait is combined with a cognitive task provides more time to adequately position the foot on the ground, thus preserving postural stability. Contrary to walking, SIP does not require a high level of regulation of dynamic stability, as the forward progression of the center of mass does not need to be controlled. Therefore, foot placement during SIP may require less active regulation than normal walking and consequently allow more of one’s cognitive resources to be allocated to the cognitive performance. As gait velocity has been shown to decrease in both fallers [34, 35] and in individuals with cognitive impairments [36–38], it would have been interesting to determine if cycle duration would decrease in these populations despite the simplicity of the SIP task. However, a larger sample size would have been necessary to further divide our older adult group.

Previous studies have reported larger gait variability in older adults when performing a Category fluency task [29], but have shown no effect in younger adults [23, 29]. Recently, Walshe et al. [22] reported a larger negative effect of dual tasking on gait, but even more so when the cognitive task targeted the executive functions. In their study, gait in both young and older adults was affected, but with a larger effect in older adults. Altogether, these studies demonstrated that the generation of the gait pattern involves higher cortical regions [22, 23, 29]. The greater effect of dual tasking on older adults also suggests that as age progresses, cognitive resources may be inadequate to perform gait and a cognitive task simultaneously [12, 22]. Overall, our results are in line with these previous studies, which demonstrate that age and dual tasking affect the generation and regulation of the SIP activity. However, SIP arrhythmicity was larger in older adults, independently of the cognitive task, while asymmetry was not affected until concomitant cognitive activity was performed. This suggests that age affects the ability to regulate stepping rhythmicity to a greater extent than it affects stepping asymmetry, and that both necessitate higher-level cognitive functions independently of the control of postural balance.

Overall, cognitive performance was affected by age both in single and dual tasking conditions, which highlights the age-related decline in executive processes performance [39]. Interestingly, both the Category task and Stroop A (words, congruent) showed a main effect for tasks while Stroop B (colours, incongruent) did not. This could be due to what was previously described by Bloem and collaborators [27] as the *posture first* strategy, whereby older adults could have been prioritizing motor performance over cognitive performance when the level of difficulty of the cognitive task was increased (incongruent vs. congruent). The positive aspect of such a strategy is that it reduces the risk of postural instability during the completion of the motor task. However, on the basis of the older adults’ decreased performance in both the motor and cognitive task compared to younger adults, these strategies may not be entirely effective. As participants in the present study were healthy older adults, it is more likely that motor and cognitive performances in individuals with cognitive deficits would have been even more affected by the dual task paradigm [34–38].

The type of cognitive task chosen is important to consider when looking at its effect on gait, as it has been demonstrated that tasks involving executive function have a larger effect on gait compared to tasks used to divide attention (e.g. reciting the alphabet) [20, 22, 40]. Both cognitive tasks performed in this study are considered to rely mainly on the executive functions. However, specific characteristics of these tasks could explain motor and cognitive discrepancies between the tasks. While verbal fluency is largely attributed to the executive functions, it has been shown to be dependent on the visuospatial sketchpad, a subsystem of the working memory, and therefore, participants may use visual imagery to retrieve items from a category [41]. In our study, despite the fact that participants were asked to fixate on a monitor during the trial, the visualization strategy could have led to an internal focus of attention. This is important when considering that the Stroop Word and Color Test necessitates focusing “externally” on the monitor. Therefore, the monitor could have played the role of a *visual anchor*, stabilizing balance and reducing stepping asymmetry by a greater extent during the Stroop Word and Color Test than during the category task.

## Conclusions

Age and the type of a cognitive task affected stepping characteristics differently. While SIP arrhythmicity was larger in all conditions in older adults compared to younger adults, SIP cycle duration was not significantly different and asymmetry showed a trend to be larger when a verbal fluency task was performed while stepping in place. This suggests that the regulation of the stepping activity relies more heavily on the executive function as age progresses and that the age related decline in these higher-level cognitive functions affects the ability to generate rhythmical stepping cycles. Considering that this study was conducted on healthy older adults, it would be interesting to assess stepping variability in older adults with cognitive decline and in individuals at high risk of falls.
